# Editorial: Bioethics in neonatology

**DOI:** 10.3389/fped.2024.1501864

**Published:** 2024-10-16

**Authors:** Ana Concheiro Guisán, Sonia Caserío Carbonero

**Affiliations:** ^1^ Instituto de Investigación Sanitaria Galicia Sur (IISGS), Vigo, Spain; ^2^ Pediatrics, Facultad de Medicina y Odontología, Universidade de Santiago de Compostela, Santiago de Compostela, Spain; ^3^ Department of Pediatrics, Alvaro Cunqueiro University Hospital, Vigo, Spain; ^4^ Neonatology Department, Hospital Universitario Río Hortega, Valladolid, Spain; ^5^ Pediatrics, Facultad de Medicina, Universidad de Valladolid, Valladolid, Spain

**Keywords:** end-of-life decision-making, limit of viability, bioethics, neonatology, quality of life

**Editorial on the Research Topic**
Bioethics in neonatology

The topic *Bioethics in Neonatology* includes five valuable articles that address the ethical complexity of certain situations that occur during neonatal care. Special reference is made to those moments where the beginning and end of life converge and decisions regarding the appropriateness of therapeutic effort (ATE) are made. Specifically, extreme prematurity at the limit of viability is mentioned. The survival rate among children born at this limit has increased in recent decades; however, the absence of long-term sequelae has not increased in parallel. The commitment to the future life expectancy and quality of life of these children is at the core of ethical decision-making.

Each of the articles has been written by different authors who diverge in their geographical context, coming from multiple cultural backgrounds. This undoubtedly enriches the topic, providing such delicate issues with a diverse, although sometimes complementary, socio-cultural perspective.

We recommend starting the reading of this collection with the article by Morillo Palomo et al. This work helps to frame the topic by providing a global reflection on the end-of-life decision-making process in Neonatology. These situations are challenging, although not exceptional; it should be remembered that one-third of pediatric deaths occur in the neonatal period, and a large part of them occur after a decision to withdraw or not to initiate life support measures in a palliative care context. The article proposes overcoming a reductionist view that limits bioethical analysis to the classical principles of bioethics proposed by Beauchamp and Childress (Beauchamp, T. L., & Childress, J. F. *Principles of Biomedical Ethics*, 1979). At the same time, it transcends the assumption that only the best interest of the child should be at the center of deliberation. Instead, the authors propose prioritizing non-maleficence and protecting the *parental discretion zone*. This zone is understood as an ethical space where parents can make legitimate decisions about their children, even if they do not fully align with what is considered the best interest for the child, always considering that they cannot inflict harm or suffering. The authors propose a shared decision-making process with the family. Sharing the process reconciles the technical dimension of the decision to be made with the values at stake. Family participation, to the extent they desire, can help them overcome grief, and for professionals, sharing the decision prevents moral distress. *Relational autonomy* is valued, placing on the table the emotional aspects of the decision-makers that are inherent to the decision-making process.

The weight of family opinion in decision-making varies according to the socio-cultural context presented in the different articles. In the article authored by Syltern, reference is made to the differences between Scandinavian countries. In these countries, depending on the established protocols for birth at the limits of viability, the active resuscitation of premature infants in the grey zone will be decided either based on the best interest of the child (Sweden) or prioritizing the family's opinion (Denmark). In the survey conducted among professionals in pediatric and neonatal intensive care units in Croatia, presented by Curkovic et al., the opinion of families in end-of-life decisions has variable weight for the different professionals surveyed. This variability is partly justified by the lower protocolization and regulation of ATE processes in this country. The authors also reflect on the possible influence of their historical-cultural background and religious beliefs on a certain level of polarization detected in other responses, such as the degree of commitment, acceptance, and/or experience of professionals in carrying out these processes.

In a different cultural context, such as countries where Islam is the majority religion, it will be the *sharia*, law extracted from religious texts, embodied in the *fatwa* or religious opinion, that will condition parental decision-making in most situations, including active care at the limits of viability. The work written by Bin Shoaib reflects on the possible ignorance or confusion that both professionals and families may have about what the sacred texts indicate about proceeding in these situations, for example, the legitimacy or not of terminating a pregnancy before or after the moment of “ensoulment” of the fetus. The author points out that the doctrinal guidelines in decision-making are compatible with the consideration of individual aspects in each particular case, placing the family, according to their faith, values, and life circumstances, at the center of the process.

Finally, this topic includes the results of a second survey of professionals presented by Wang et al. It is a study involving 31 healthcare centers in China and more than 2,300 respondents, focusing on finding differences in establishing the limit of viability between obstetricians and neonatologists. Most neonatologists set the limit around Western standards (24 weeks), while obstetricians define another gestational limit (28 weeks) that countries in other geographical areas have surpassed decades ago; in these countries, the debate is now centered on a grey zone between 22 and 24 weeks. It is of interest to analyze the different perspectives of professionals, considering the influence that the professional providing information to the family may have on decision-making. It is noteworthy that the opinion of families has a determining weight in the decision of active resuscitation of the premature infant for both surveyed groups, along with their weight and gestational age. This work once again highlights the importance of protocolization and legislation of end-of-life and ATE processes to ensure homogeneous, equitable, and excellent care.

We recommend an in-depth reading of this topic, valuing its multicultural perspective, although some contexts, such as Latin American, may be missing. In one way or another, the content of all the articles contributes to highlighting the importance that, in shared decisions, knowing, respecting, and assuming the values and cultural and/or religious context of families inevitably has. In a globalized world like the one we share, reading this topic can undoubtedly help professionals facing these situations to approach them successfully, helping to understand the context experienced by families from different backgrounds. It is essential to protocolize and at the same time personalize end-of-life decision-making in Neonatology to offer compassionate care, centered on the family, that also takes care of the emotional well-being of all parties involved, including professionals.

